# Exergaming for Healthy Aging: Associations with Functional Capacity, Social Participation, Self-Efficacy for Exercise, and Adherence to Inform Exergame Development

**DOI:** 10.3390/s26144616

**Published:** 2026-07-21

**Authors:** João Quatorze, Magda Reis, Guilherme Alvarez, Anabela Correia Martins

**Affiliations:** 1 Coimbra Health School, Polytechnic University of Coimbra, Rua 5 de Outubro, 3045-043 Coimbra, Portugal; joao.quatorze@physio14.ch (J.Q.); a2021130990@estesc.ipc.pt (M.R.); a2017070285@estesc.ipc.pt (G.A.); 2 Physio14, Route de Saint-Légier 15A, 1800 Vevey, Switzerland; 3 Fisioterapia, Centro Cirúrgico de Coimbra, R. Dr. Manuel Campos Pinheiro 51, 3045-089 Coimbra, Portugal; 4 H&TR-Health & Technology Research Center, Coimbra Health School, Polytechnic University of Coimbra, Rua 5 de Outubro, 3045-043 Coimbra, Portugal; 5 Center for Rehabilitation Research, Health School, Polytechnic of Porto, Rua Dr. António Bernardino de Almeida, 4200-072 Porto, Portugal; 6 SUScita—Research Group on Sustainability, Cities and Urban Intelligence, 3045-093 Coimbra, Portugal

**Keywords:** FallSensing, exergaming, sensors, Otago Exercise Program, older adults

## Abstract

This study explores current applications of exergaming in healthcare, with a focus on how the Otago Exercise Program—a structured, evidence-based program designed to improve strength and balance in older adults—integrated into the FallSensing exergames, contributes to improving older adults’ functioning. It also aims to generate evidence to support the optimization of sensor-based technologies for more personalized and adaptable exercise interventions. Community-dwelling older adults (≥60 years) were recruited from facilities in Coimbra, Portugal, and allocated into an exergames group (IG; *n* = 27) and a control group (CG; *n* = 34). The CG maintained usual daily activities, while the IG completed an 8-week (16-session) exergame-based program. After completing the program, the CG showed a decline in functional ability, whereas the IG demonstrated significant improvements in the Step Test (*p* = 0.001), Four-Stage Balance Modified Test (*p* = 0.001), Self-Efficacy for Exercise Scale (*p* = 0.009), and Activities and Participation Profile Related to Mobility questionnaire (*p* < 0.001). Exergaming was safe and effective in enhancing functional ability, participation, and self-efficacy in older adults. However, careful consideration of exercise frequency, intensity, and participants’ age is recommended when prescribing exergame-based interventions. These results also highlight another interesting topic among physiotherapists who prescribe and monitor exergames, that technology developers should consider exercise-time monitoring systems that integrate physical (e.g., eye, facial, and mouth features) and physiological signals to enhance fatigue detection accuracy.

## 1. Introduction

According to the United Nations [1], the global population aged 60 years or older was approximately 962 million in 2017, representing 13% of the total population, with Europe having the highest proportion. Rapid population aging is expected worldwide, and by 2050 nearly all regions—except Africa—will have about one in four people aged 60 or older. Currently, this age group numbers about 1.2 billion, projected to rise to 1.4 billion by 2030 and 2.1 billion by 2050, representing roughly 22% of the global population [2,3,4].

As the body ages, cellular and systemic changes occur that can impair physical and cognitive functions, often reducing independence and quality of life [5]. Biologically, aging involves the gradual accumulation of cellular and molecular damage, leading to functional decline, increased vulnerability to environmental stressors, and a higher risk of disease and mortality [6].

Motor functions are strongly affected by aging. Reductions in skeletal muscle mass and strength, together with changes in muscle contractile properties, impair motor performance. These alterations also contribute to decreased bone mass and mechanical strength, increasing the risk of fractures related to osteopenia and osteoporosis [7,8]. Additionally, degeneration of articular cartilage and reduced synovial fluid volume lead to joint stiffness and fragility, limiting range of motion and, in some cases, causing pain [9].

These mobility limitations make it difficult for older adults to maintain independence in activities of daily living (ADLs) and to participate socially. Simple tasks, such as rising from a chair or crossing a street, can become challenging or even impossible [8,10]. Reduced walking speed is commonly observed and is one of the strongest predictors of adverse outcomes in older adults [9,11]. Gait speed is also a well-established indicator of functional decline and mortality risk in this population [12,13].

Aging is also associated with sensory and cognitive changes. Hearing and vision impairments can limit mobility and increase the risk of falls [14,15]. Other common age-related changes include declines in memory and information processing speed, as well as alterations in sexual, neurological, endocrine, cardiopulmonary, gastrointestinal, urinary [5,16], and immune system functions [17].

Exercise is an effective strategy for preventing and reducing age-related functional decline, as well as a therapeutic intervention to counteract the detrimental effects of aging [10,18]. However, traditional exercise can be less engaging for older adults, and exergaming has emerged as a promising alternative.

Exergames are computer games that are driven by gross physical movements of the player. Also called “virtual reality-enhanced exercises”, exergames are currently undergoing academic research as they become popular as a means to promote healthy behaviors and increase the appeal of exercise [19,20]. Metaphorically, exergames are a kind of “mousetrap” that increases physical activity. This type of exercise shifts attention away from aversive aspects of exercising and can be undertaken at home, making it more accessible. Thanks to features such as competition and three-dimensional scenery, this option motivates all, including the older ones, to participate in such programs [21].

Adapting traditional exercise programs into video games creates engaging, sensory-stimulating experiences, and evidence suggests that exergaming can sometimes be more effective than conventional exercise alone [3]. Exergames have been shown to provide multiple benefits, including health promotion and disease prevention [22], improved physical performance [23], enhanced social well-being—such as reduced loneliness, increased social connection, and more positive attitudes toward others [18,24,25]—greater physical activity and self-efficacy [26,27], improved perceived wellness [28], better balance and gait [29,30,31,32,33], increased strength [34], enhanced cognitive function and dementia prevention [35,36], positive emotional experiences [37], higher adherence to exercise programs [38,39], and reduced fear and risk of falling [40,41,42].

Several solutions have been developed that harness the entertainment value of gaming to increase physical activity and exercise, improving health outcomes [19,43]. However, most existing solutions were designed for individual use or for only two players. To the best of our knowledge [44], this is the first exergaming solution to incorporate the well-established P (OEP) in a group-based playing format.

The OEP is an evidence-based, individually tailored strength and balance training program for older adults, shown to significantly reduce the risk of falls and related injuries. The original program, developed at the University of Otago Medical Sciences, includes five strengthening exercises and twelve balance exercises, performed three times per week. Participants are also instructed to perform aerobic exercises, such as walking for 30 min twice a week [45]. Previous research has demonstrated the program’s effectiveness: a 2010 systematic review and meta-analysis found that the OEP significantly reduces the risk of death and falls among community-dwelling adults aged 65 and older [46], and a 2017 study reported a 35% reduction in the number of falls among participants [47]. Additionally, the OEP has been shown to reduce fear of falling (FOF) when performed more than twice a week for at least 24 weeks in community or elderly care settings [40]. Despite its proven benefits, adherence to traditional exercise programs in older people is often low, suggesting the need for new, and more engaging approaches [39]. In this study, the exercises included in the OEP were implemented through an exergame format designed to enhance motivation and adherence.

This study aims to explore how participation in these exergames may influence indicators of health and functioning, including functional capacity, social participation, exercise self-efficacy, and adherence, in comparison with individuals who do not engage in structured exercise. Given that exergames rely heavily on sensors to capture and interpret users’ movements, evaluating their implementation, clinical applicability, and limitations may provide important insights into the potential and challenges of this emerging technological approach, without intending to establish causal inferences.

## 2. Materials and Methods

### 2.1. FallSensing Exergames

**Figure 1:**
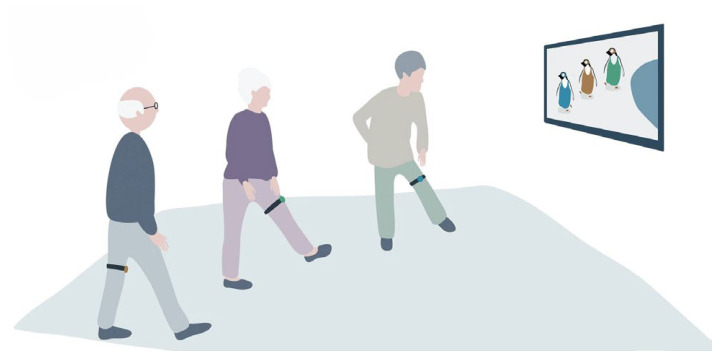
Illustration of the game design based on an Antarctic theme, where penguins are featured as the main characters.

The FallSensing exergames (Sensing Future Technologies, Coimbra, Portugal, and Fraunhofer Portugal AICOS, Oporto, Portugal) comprise three mini games designed to be played by two teams, each consisting of up to three players competing alternately. Participants wore inertial sensors that captured and monitored movement execution. The sensor is positioned on the thigh, ankle, or upper foot, depending on the specific game. Each mini-game features distinct rules and gameplay dynamics determined by the required movement, which is based on a set of exercises from the OEP. The movements to be performed are repeatedly explained to ensure their safe and correct execution. The system processes the movement data, which are then used to animate the player’s avatar. Integration of OEP exercises into the mini games followed the principle of grouping exercises that require identical sensor placement on the body. This strategy minimizes the need for repositioning the sensor, supports gameplay continuity, and balances the relative physical effort across exercises. The spatial arrangement, including the distance between players and between the players and the display, was also taken into consideration. The game was designed around an Antarctic theme, with penguins serving as the main characters (Figure 1). Penguins were selected as avatars because they can be modelled to perform the required exercises effectively while introducing an element of enjoyment and engagement for users, in part, due to their funny walk. This system differentiates between two user types: players and the administrator. The players are older adults who perform prescribed physical exercises while wearing the designated sensor. The game administrator is responsible for initializing the system on a computer and assigning players to their respective teams—a role that may also be fulfilled by one of the players. During gameplay, participants preferably face a television screen displaying the game interface and wear a strap on their lower limbs containing an inertial measurement unit, a color-coded sensor corresponding to the penguin avatar’s color. This wearable device enables real-time motion detection and characterization, thereby triggering in-game actions and facilitating subsequent analysis of each participant’s performance [48].

Following the development of exergames, and preliminary tests, it was essential to evaluate them with real user populations to determine their clinical relevance and user acceptance. Despite their value in early-stage design, controlled laboratory assessments provide a limited representation of real-world interactions and behavioral responses in older adults. Empirical studies conducted with representative cohorts yield critical evidence related to usability, engagement, safety, and potential therapeutic benefits [49]. Moreover, such evaluations ensured that the system not only achieves its intended clinical and functional goals but also meets the needs, preferences, and motivational profiles of its end users—factors that are crucial for long-term adherence, satisfaction, and effectiveness in real-life contexts.

### 2.2. Study Design

Participants were recruited from several community settings between June and December 2018. This quasi-experimental study employed a non-randomized allocation procedure following initial screening conducted within the framework of the FallSensing Project. Participants aged 60 years or over were invited to participate in the study after they finished the fall risk screening. As the screening protocol required participants to be able to stand and walk independently, with or without walking aid, all individuals potentially enrolled met these criteria. Although individuals with a history of cerebrovascular or neurological disorders, severe sensory impairments (deafness or blindness), or cognitive impairments were allowed to participate in the fall risk screening, they were not invited to the study because such conditions could compromise their ability to understand and complete the questionnaires and functional assessments required during the evaluation phase. Allocation was performed by three physiotherapists who were members of the research team but were not involved in the delivery of the exergame intervention, using a convenience sample. The intervention was subsequently implemented by a separate team within the project.

Upon completion of the 8-week intervention period, all participants were reassessed using the same procedures applied at baseline.

Ethical approval was obtained from the Ethics Committee of the Polytechnic Institute of Coimbra (registration code: 6_CEIPC_2017). All participants provided written informed consent prior to data collection, in accordance with the Declaration of Helsinki. The study was registered on ClinicalTrials.gov (Identifier: NCT03623919). Participant anonymity and data confidentiality were strictly maintained. The study was conducted solely for scientific purposes, and no conflicts of interest are declared.

### 2.3. Participants

For inclusion in the present study, participants had to be aged 60 years or older and express interest in participating after the invitation. Participants who met the inclusion criteria but were unable to attend the intervention sessions with the frequency required for participation in the program were allocated to the control group. Nevertheless, all agreed to return to the study site 8 weeks later to repeat the assessment procedures.

### 2.4. Description of Intervention

#### 2.4.1. Exergames Group

Intervention involved the OEP incorporated in the FallSensing Exergames, with pressure and inertial sensors. Participants had a total of 16 sessions, for 8 weeks, approximately two times a week, lasting for about 20 min. This exercise program targeted the knee flexors and extensors, hip abductors, ankle dorsi flexor/plantar flexor muscles and balance.

Participants did not receive any physical assistance during the exergaming sessions. Sessions were supervised for safety purposes, with verbal feedback provided to correct movements, when necessary, as well as technical assistance related to sensor placement and repositioning to ensure proper system functioning. This procedure is consistent with the intended use of exergaming interventions, which are typically implemented under supervision to ensure safety and appropriate interaction with the system. Participants were required to attend at least 90% of the 16 sessions delivered over the 8-week intervention period to be included in the analysis.

Adverse events, including falls, near falls, pain, dizziness, discomfort, and any other unusual symptoms, were monitored throughout the intervention period and/or assessed at the end of each exergaming session. All events were documented when observed during the sessions or when spontaneously reported by participants.

#### 2.4.2. Control Group

The control group comprised individuals who did not participate due to lack of interest or unavailability and therefore received no intervention. They were encouraged to maintain their usual daily routines.

### 2.5. Outcomes

Individual assessments were made at baseline (T0) and after 8 weeks of undergoing intervention (T1), by trained physiotherapists.

The outcome measures were part of the FallSensing screening protocol, which included both questionnaires (sociodemographic and clinical data), self-efficacy for exercise and social participation profile, and standardized tests [50].

#### 2.5.1. Questionnaires

The Self-Efficacy for Exercise Scale (SEE) is a five-item questionnaire used to assess the participants’ confidence in performing physical activity under five emotional conditions: feeling worried or experiencing problems, feeling depressed, feeling tired, feeling tense, and being busy [51].

The Activities and Participation Profile Related to Mobility (PAPM) is an 18-item instrument, originally developed in Portugal, designed to assess the extent of difficulty individuals experience in performing daily activities in their natural environment. It covers domains such as social interactions, education, employment, financial management, and community life, all of which may influence social participation. Responses are rated on a 5-point Likert scale ranging from 0 (“no limitation or restriction”) to 4 (“complete limitation or restriction”). Not all items are applicable to every individual, and ‘NA’ (not applicable) should be marked when appropriate. A participation profile is generated based on valid responses, and the final score should be interpreted along a continuum of restriction levels. Scores from 0 to 0.19 indicate no restrictions on participation. Values between 0.20 and 0.99 reflect mild restrictions, while scores between 1.00 and 1.99 denote moderate restrictions. Scores between 2.00 and 3.87 represent severe restrictions, and scores between 3.88 and 4.00 correspond to complete restrictions on participation [52].

#### 2.5.2. Functional Tests

Participants performed the handgrip strength (HS) test while seated in a standard armless chair, with shoulders adducted and neutrally rotated, elbows flexed at 90°, and wrists positioned in 0–15° of ulnar deviation. A hand dynamometer was used, set to the second handle position and held in the dominant hand in a vertical orientation. Participants were instructed to exert maximal grip strength for 5 s. The result was recorded in kilograms of force (kgf). Normative reference values differ by sex; values below 15 kg in women and below 21 kg in men indicate an increased risk of falls [53]. In the Portuguese population, the mean grip strength was 24.2 kg (SD = 8.82).

Gait speed was measured along a 20 m walkway, with the initial and final 5 m designated for acceleration and deceleration by the 10-Meter Walk Speed (10-mWS) test. Timing was recorded between the 5 and 15 m marks. Participants wore comfortable footwear and were allowed to use assistive devices. Walking speed ≤ 1 m/s indicates higher fall risk, whereas speed ≥ 1.42 m/s is considered sufficient for safe street crossing [54]. In the Portuguese population, the mean walking speed was 1.44 m/s (SD = 0.43).

The 30 s Sit-to-Stand (30 s STS) test evaluates lower limb strength by counting the number of times participants can rise from a seated to a standing position and return to sitting within 30 s. The final score corresponds to the total number of complete repetitions performed. Normative values differ according to age and sex [55]. In the Portuguese population, the mean number of repetitions in the 30 s STS test was 11.6 (SD = 4.09).

The Timed Up and Go (TUG) test measures dynamic balance, mobility, and lower limb strength. Participants started seated in a standard chair and were instructed to stand up, walk 3 m, turn around, return to the chair, and sit down as quickly and safely as possible, without running or using upper limb support. A completion time greater than 12 s indicates an increased risk of falls in older adults [56]. In the Portuguese population, the mean time to complete the Timed Up and Go test was 9.84 s (SD = 6.43).

The Step Test (ST) was designed to assess dynamic standing balance and reproduce lower extremity motor control and coordination. Participants are asked to step on and off a block (7.5 cm height, 55 cm width, and 35 cm depth) placed against a wall as many times as possible for a duration of 15 s. Participants are unsupported and should look straight forward, although the test administrator must stand close by for safety. This test is performed only for the dominant side, as indicated by the person being tested. The performance of <10 steps indicate a higher risk of falling [50].

The Four-Stage Balance “modified” (4StageBTM) test assesses balance. To perform this test, the participant needs to progressively accomplish 4 different foot positions: side-by-side stance, semi-tandem stance (preferred foot forward with the instep of one foot touching the big toe of the other foot), tandem stance (one foot in front of the other, heel touching toe), and one-legged stance (preferred leg for support). The participant is instructed to stand quietly, arms along the body, with neither shoes nor assistive devices. Positions must be held for 10 s each without moving the feet, needing support, losing balance or touching the leg of support with the other leg and must be performed with eyes open and then closed (excluding one-legged stance position). The sequence will be side-by-side stance with eyes open, side-by-side stance with eyes closed, semi-tandem stance with eyes open, semi-tandem stance with eyes closed, tandem stance with eyes open, tandem stance with eyes closed, and one-leg stand with eyes open. If the person fails to accomplish one of the test positions, the test finishes. The final score will be the number of positions that are successfully completed. The inability to complete 10 s in the tandem stance position with eyes open has been associated with a higher risk of falling and mobility dysfunction [50].

The selected outcomes reflect the multidimensional nature of functioning in older adults, encompassing physical capacity, behavioral engagement, and psychosocial factors. Functional measures capture physical performance, while self-efficacy, adherence, and social participation provide insight into motivation, sustainability, and real-life impact of the intervention. Together, these domains allow a more comprehensive evaluation of the potential benefits of exergame-based interventions.

### 2.6. Statistical Analysis

After the data collection phase, data was entered into Excel 2016 (Microsoft Office, Redmond, WA, USA) and statistical data analysis was completed using IBM Statistical Package for the Social Sciences (SPSS), version 24. Values of *p* < 0.05 were counted as statistically significant.

In the sample characterization, descriptive statistics were used, such as measures of central tendency (mean, M) and dispersion (standard deviation, SD). Missing data was excluded from the analyses.

Differences between the control and exergames groups were initially assessed using independent-samples Student’s *t*-tests. To further account for potential confounding due to age and baseline group differences, analyses of covariance (ANCOVA) were performed for each post-intervention outcome, including age and the corresponding baseline measure as covariates. Adjusted group means (estimated marginal means, EMMeans), adjusted between-group differences with 95% confidence intervals, F statistics, *p* values, and partial eta-squared (η^2^*p*) are reported for all ANCOVA models. Statistical significance was set at *p* ≤ 0.05. In addition, between-group effect sizes (Hedges’ g) were calculated at baseline, post-intervention, and for changes over time (difference-in-differences) to quantify the magnitude of the observed effects.

## 3. Results

Eighty-seven individuals from three different day care centers expressed interest in participating. Permission was obtained from these facilities. Of the 87 potential participants, 61 adults aged 60 years or older provided informed consent and were included in the study. Participants were then divided into two groups according to their willingness to participate in the adapted exergames program.

Table 1 presents baseline characteristics of the 61 participants. In this study sample, 77% of the participants were female. The mean ages of the exergames group (EG) and control group (CG) were 82.22 (SD = 8.290) (ranging from 65 to 92 years) and 87.26 (SD = 5.812) years (ranging from 77 and 99 years), respectively. In terms of body mass index (BMI), the global sample has a mean value of approximately 27.3 kg/m^2^, corresponding to being “overweight”. BMI values ranged from 18.07 to 39.64 kg/m^2^.

At baseline measurements, PAPM had a mean value of 1.670 with an SD of 1.112 and SEE had a mean value of 12.47 with an SD of 5.117.

Baseline and post-intervention outcomes, including intergroup and intragroup analyses, are presented in Table 2. Overall, baseline comparability between groups was confirmed, with no statistically significant differences observed for most variables. However, significant differences were identified for the 4StageBTM (*p* = 0.020) and the 10-Meter Walk Test (*p* = 0.030), which were considered in the interpretation of subsequent results.

After 8 weeks, the control group exhibited an overall decline in functional performance. The most pronounced deterioration was observed in the TUG, which increased from 19.395 ± 12.816 to 23.914 ± 18.616 s (*p* = 0.064). Minor declines were also observed in handgrip strength (HS) (13.76 ± 5.830 to 13.55 ± 5.731 kgf; *p* = 0.636), gait speed (10-MWT: 0.429 ± 0.350 to 0.422 ± 0.286 m/s; *p* = 0.853), and lower limb strength (30 s Sit-to-Stand: 7.150 ± 4.966 to 6.55 ± 4.359 repetitions; *p* = 0.293).

Conversely, small changes were noted in dynamic balance (Step Test: 6.11 ± 4.310 to 6.50 ± 3.823 repetitions; *p* = 0.565) and static balance (4StageBTM: 2.95 ± 1.786 to 3.14 ± 1.699 positions; *p* = 0.358), although these changes were not statistically significant.

Regarding participation and psychosocial outcomes, participants reported a decline in activity performance (PAPM: 1.466 ± 1.006 to 1.744 ± 0.902 points; *p* = 0.275). Exercise self-efficacy (SEE) showed a negligible increase (12.75 ± 4.898 to 12.85 ± 4.705 points; *p* = 0.927), which was not statistically significant.

The exergaming group demonstrated consistent changes across all functional measures. Statistically significant gains were observed in dynamic balance (ST: 5.65 ± 2.412 to 8.10 ± 3.024 repetitions; *p* = 0.001) and static balance (4StageBMT: 3.56 ± 1.368 to 4.70 ± 1.235 positions; *p* = 0.001).

Although not reaching statistical significance, changes were also found in 10-MWT (0.734 ± 0.325 to 0.860 ± 0.305 m/s; *p* = 0.112), lower limb strength (30s STS: 7.89 ± 5.235 to 9.30 ± 3.473 repetitions; *p* = 0.141), TUG (20.325 ± 11.772 to 18.319 ± 12.880 s; *p* = 0.103), and HS (15.24 ± 8.797 to 15.52 ± 6.226 kgf; *p* = 0.847).

Significant favorable changes were also observed in psychosocial and participation outcomes, with PAPM scores decreasing from 2.398 ± 1.069 to 0.999 ± 0.574 (*p* < 0.001), indicating improved participation, and SEE increasing from 12.15 ± 5.082 to 14.74 ± 4.469 (*p* = 0.009).

### 3.1. Effect Size Analysis

Effect size analysis using Hedges’ g revealed consistent between-group differences at post-intervention, favoring the exergaming group across multiple domains.

Large effect sizes were observed for walking speed (10-MWT; g = 1.47, 95% CI [0.90, 2.04]) and static balance (4StageBMT; g = 1.02, 95% CI [0.48, 1.56]), indicating substantial intervention-related improvements. A moderate-to-large effect was found for lower limb functional strength (30s STS; g = 0.68, 95% CI [0.16, 1.20]). Small-to-moderate effects were identified for dynamic balance (ST; g = 0.45, 95% CI [−0.06, 0.96]) and exercise self-efficacy (SEE; g = 0.41, 95% CI [−0.11, 0.92]). A small effect was observed for functional mobility and balance (TUG; g = −0.34, 95% CI [−0.85, 0.17]), suggesting a modest benefit in favor of the exergaming group. A small effect was also observed for handgrip strength (HS; g = 0.33, 95% CI [−0.18, 0.84]). Additionally, a large effect size was found for participation (PAPM; g = −0.95, 95% CI [−1.48, −0.42]), highlighting a substantial impact of the intervention beyond physical performance.

Overall, the observed pattern of variances suggests that exergaming may have potential clinical relevance across functional and psychosocial outcomes; however, some confidence intervals crossed zero, indicating greater uncertainty for specific outcomes.

In parallel with the results observed in functional capacity, social participation, and exercise self-efficacy, physiotherapists reported favorable emotional experiences and a positive attitude among participants. Adherence in the exergames group was nearly complete, with all participants completing the full 16-session program except for one participant, who completed 15 sessions.

### 3.2. Adjusted ANCOVA Analyses

ANCOVA models adjusting for age and the corresponding baseline value of each outcome were performed for all post-intervention outcomes. Adjusted group means (estimated marginal means), adjusted between-group differences with 95% confidence intervals, F statistics, *p* values, and partial eta-squared effect sizes are presented in Supplementary Table S1.

Following adjustment, statistically significant between-group differences were observed for the Timed Up and Go test (*p* = 0.007), 4StageBTM (*p* = 0.011), 30 s Sit-to-Stand test (*p* = 0.045), and Participation Index (*p* = 0.002), whereas no statistically significant adjusted differences were observed for grip strength (*p* = 0.866), walking speed (*p* = 0.089), Step Test (*p* = 0.063), or exercise self-efficacy (*p* = 0.137).

Overall, adjustment for age and baseline performance attenuated the between-group differences for several outcomes, highlighting the importance of accounting for baseline imbalances when interpreting the findings.

## 4. Discussion

The present study found that participation in an exergaming-based program was feasible and was not associated with adverse events during the intervention period. Participants in the exergaming group also demonstrated favorable changes in functional capacity, social participation, and exercise self-efficacy over the 8-week intervention. However, these findings should be interpreted cautiously because this was a pragmatic quasi-experimental study with non-randomized group allocation and baseline differences between groups. Consequently, the observed between-group differences cannot be interpreted as evidence that the intervention itself caused the observed changes, but rather associations that warrant confirmation in adequately powered randomized controlled trials.

### 4.1. Functional Capacity

For analytical clarity, functional outcomes were grouped into strength (HS and 30s STS) and balance (ST, 4StageBMT and TUG), with walking speed (10-MWT) considered separately.

#### 4.1.1. Strength and Mobility

Participants in the control group, who maintained their usual daily activities, showed a general decline in strength-related outcomes over the 8-week period, whereas participants in the exergaming group showed changes in HS and 30s STS, although not all changes reached statistical significance.

The reduction in standard deviation observed in HS and 30s STS suggests less variability in performance within the intervention group over time. Although this pattern may indicate more homogeneous functional performance, it should be interpreted cautiously given the study design and sample size.

The 8-week intervention may have been insufficient to produce maximal physiological adaptations, particularly muscle hypertrophy. Previous evidence indicates that hypertrophic adaptations in older adults generally require interventions lasting at least 12 weeks, with higher training frequency and greater resistance stimuli [57,58,59]. Nevertheless, shorter interventions have been associated with neuromuscular adaptations [60,61,62,63], providing a possible explanation for the functional improvements observed. However, because of the quasi-experimental design, these findings cannot establish that the intervention was responsible for the observed changes.

Comparisons between baseline and follow-up showed variations in HS, 30s STS, and TUG in the exergaming group, with larger changes observed in 30s STS and TUG than in HS. One possible explanation is that these measures include dynamic balance and functional mobility components that may be more responsive over shorter periods than isolated strength measures. However, alternative explanations, including baseline differences, regression to the mean, and unmeasured confounding, cannot be excluded.

#### 4.1.2. Balance Performance

Balance-related outcomes showed the most consistent changes in the exergaming group, with statistically significant changes observed in ST, 4StageBMT and TUG.

These findings are consistent with previous studies suggesting that balance measures are often more responsive than strength measures during relatively short exercise interventions [64,65,66]. Participants in the exergaming group improved their ST performance from 5.65 to 8.10 repetitions and progressed in the 4StageBMT toward values approaching normative data for community-dwelling older adults [67]. The control group, in contrast, generally showed stable or declining balance performance over the same period. Although these patterns are compatible with previous literature, they should not be interpreted as causal effects of the intervention because differences between groups may also reflect baseline characteristics, age differences, or other uncontrolled factors [68,69,70,71,72,73].

#### 4.1.3. Walking Speed

Walking speed is a global marker of functional health that integrates lower-limb strength, motor control, and postural stability [54,74]. While the control group showed minimal change, the exergaming group demonstrated a non-significant improvement in walking speed. Although post-intervention values continued to indicate some degree of mobility limitation, the direction of change is consistent with previous exergaming studies reporting similar trends [32,75]. Given the study design, these findings should be regarded as exploratory and hypothesis-generating rather than confirmatory evidence of intervention effectiveness.

### 4.2. Social Participation

The exergaming group demonstrated statistically significant improvements in participation scores, whereas the control group showed a worsening trend over time. These findings are consistent with previous reports suggesting that exergaming may be associated with improved confidence and participation in daily activities among older adults [24,69,76,77]. However, because participants were not randomly allocated and groups differed at baseline, it is not possible to conclude that the intervention itself was responsible for these improvements.

Participants initially presented severe participation restrictions, and post-intervention scores in the exergaming group corresponded to mild restriction according to the instrument classification. Although this represents a clinically relevant change, confirmation in randomized studies with larger samples is required before causal inferences can be made.

### 4.3. Self-Efficacy for Exercise

No baseline differences in exercise self-efficacy were observed between groups. Following the intervention period, participants in the exergaming group demonstrated higher self-efficacy scores, particularly regarding maintaining physical activity despite fatigue, worry, or low mood.

These findings are consistent with previous studies reporting improvements in exercise self-efficacy following exergaming interventions [24,78,79]. Increased enjoyment, engagement, and immediate feedback may represent plausible mechanisms underlying these observations; however, the present study was not designed to evaluate such mechanisms directly. Given the pragmatic quasi-experimental design, non-randomized allocation, and baseline imbalances, the observed improvements should be interpreted as associations rather than evidence of a causal intervention effect.

### 4.4. Game Software

Regarding the game software, participants highlighted the design, the possibility of “wearing the avatars’ skin” and competition between colleagues as contributors to a positive emotional experience, also reported in another study [80]. Some suggested the inclusion of more games, mentioning that ‘this way they would not mind exercising.’ In general, the games were understandable to most participants, although all sessions were attended by a physiotherapist who, in addition to making occasional postural and movement corrections, assisted when the game commands were not immediately understood. The polar environment of the games and the narrative structure were evaluated positively and, contrary to initial expectations, participants did not suggest changes to the game environment or the creation of more realistic avatars. Throughout the sessions, an increase in ease of execution was observed, accompanied by a reduction in the need for guidance from physiotherapists. These professionals also identified areas for improvement in terms of the comprehensibility of the games, including recording of the exercises performed, and their duration. They also pointed out that, because the games were based on the OEP, it was possible to adapt performance to the individual functional abilities of each participant with detailed explanations of how to perform the movements.

Other suggestions made by physiotherapists were that, if possible, exergames should include haptic stimuli, which is corroborated by other studies that concluded that these stimuli are preferred to auditory and visual feedback [81]. Regarding auditory feedback, it was also suggested that it could be optimized, as there are specific requirements for auditory feedback and music in exergames [82].

In future research, it would be interesting to assess the impact of different types of feedback to the body on feelings of belonging to the game, as well as on aspects related to movement control [83].

### 4.5. Limitations

Several limitations should be considered when interpreting these findings. First, this was a pragmatic quasi-experimental study with non-randomized allocation, which increases the potential for selection bias, residual confounding, and regression to the mean. Baseline differences in age and functional measures may therefore have contributed to the observed between-group differences independently of the intervention.

Second, the relatively short intervention duration (8 weeks) may not have been sufficient to elicit maximal physiological adaptations or to determine whether the observed improvements would be maintained over time. Follow-up assessments were not available, limiting conclusions regarding longer-term outcomes.

Third, participants represented a particularly vulnerable population with advanced age and multiple health-related factors that are inherently difficult to control, including comorbidities, cognitive decline, and variations in functional reserve [84]. These characteristics may also have influenced responsiveness to the intervention and limit generalizability.

Fourth, although three independent evaluators performed the assessments, blinding procedures were not implemented, introducing the possibility of observer bias.

Finally, all intervention sessions were supervised by physiotherapists. While this has likely contributed to participant safety and adherence, it may also limit the transferability of these findings to less supervised or routine community settings.

Taken together, these limitations indicate that the observed findings should be interpreted as preliminary evidence generated under real-world pragmatic conditions rather than definitive evidence of intervention effectiveness. Future studies employing randomized allocation, longer follow-up, intention-to-treat analyses, blinded outcome assessment, and larger samples are needed to determine whether the observed associations reflect true causal effects.

## 5. Conclusions

Older adulthood is frequently accompanied by reductions in physical activity, functional capacity, and social participation. Within this context, sensor-based exergaming represents a potentially valuable approach for delivering structured exercise in community settings.

In this pragmatic quasi-experimental study, participation in the exergaming program was feasible, and no adverse events were observed during the intervention period. Participants allocated to the exergaming group demonstrated favorable changes in functional capacity, social participation, and exercise self-efficacy over 8 weeks, whereas the control group generally showed stable or declining performance.

However, because of the non-randomized design, baseline imbalances between groups, and other methodological limitations, these findings should be interpreted as associations rather than evidence of causal intervention effects. The results provide preliminary support for the feasibility and potential clinical value of sensor-based exergaming in older adults with functional limitations, while highlighting the need for adequately powered randomized controlled trials with longer follow-up to determine the effectiveness of this approach and establish causal relationships.

## Figures and Tables

**Table 1:** Baseline sample characteristics.

		N	Minimum	Maximum	Mean	Standard Deviation
Age *	EG	27	65	92	82.22	8.290
	CG	34	77	99	87.26	5.812
BMI **		60	18.07	39.64	27.318	4.639
**HS**		61	4	42	13.89	6.531
**10-MWT**		34	0.11	1.50	0.578	0.3556
**30s STS**		46	1	20	7.80	4.924
**TUG**		43	6.43	65.00	20.026	11.720
**ST**		48	1	18	6.02	3.323
**4-StageBMT**		57	1	7	3.14	1.517
**SEE**		60	5	20	12.47	5.117
**PAPM**		45	0.00	3.60	1.670	1.122

BMI: body mass index (kg/m^2^); HS: handgrip strength; 10-MWT: 10-Meter Walking Speed; 30s STS: 30 Seconds Sit to Stand; TUG: Timed Up and Go; ST: Step Test; 4StageBMT: Four-Stage Balance “Modified” Test; SEE: Self-Efficacy for Exercise Scale; PAPM: Activity and Participation Profile Related to Mobility. * Intergroup age differences are statistically significant (*p* = 0.007). ** Intergroup BMI differences were not statistically significant (*p* = 0.621).

**Table 2:** Intragroup differences between baseline and post-intervention and between control and exergames groups. Values of *p* < 0.05 are shown in bold.

		Control Group (*n* = 34)	Exergames Group (*n* = 27)	*p*
HS	Baseline, mean (SD)	13.76 ± 5.830	15.24 ± 8.797	0.312
	Post-intervention, mean (SD)	13.55 ± 5.731	15.52 ± 6.226	0.291
	Difference	−0.214	0.286	
	*p*	0.636	0.847	
10-MWT	Baseline, mean (SD)	0.429 ± 0.350	0.734 ± 0.325	**0.030**
	Post-intervention, mean (SD)	0.422 ± 0.286	0.860 ± 0.305	**0.005**
	Difference	−0.006	0.126	
	*p*	0.853	0.112	
30s STS	Baseline, mean (SD)	7.150 ± 4.966	7.89 ± 5.235	0.531
	Post-intervention, mean (SD)	6.55 ± 4.359	9.30 ± 3.473	**0.016**
	Difference	−0.600	1.407	
	*p*	0.293	0.141	
TUG	Baseline, mean (SD)	19.395 ± 12.816	20.325 ± 11.772	0.839
	Post-intervention, mean (SD)	23.914 ± 18.616	18.319 ± 12.880	0.095
	Difference	4.518	−2.005	
	*p*	0.064	0.103	
ST	Baseline, mean (SD)	6.11 ± 4.310	5.65 ± 2.412	0.769
	Post-intervention, mean (SD)	6.50 ± 3.823	8.10 ± 3.024	0.159
	Difference	0.389	2.450	
	*p*	0.565	**0.001**	
4StageBMT	Baseline, mean (SD)	2.95 ± 1.786	3.56 ± 1.368	**0.020**
	Post-intervention, mean (SD)	3.14 ± 1.699	4.70 ± 1.235	**0.001**
	Difference	0.182	1.148	
	*p*	0.358	**0.001**	
SEE	Baseline, mean (SD)	12.75 ± 4.898	12.15 ± 5.082	0.667
	Post-intervention, mean (SD)	12.85 ± 4.705	14.74 ± 4.469	0.223
	Difference	0.100	2.593	
	*p*	0.927	**0.009**	
PAPM	Baseline, mean (SD)	1.466 ± 1.006	2.398 ± 1.069	0.221
	Post-intervention, mean (SD)	1.744 ± 0.902	0.999 ± 0.574	**0.019**
	Difference	0.277	−1.400	
	*p*	0.275	**<0.001**	

SD: standard deviation; HS: handgrip strength; 10-MWT: 10-Meter Walking Speed; 30s STS: 30 Seconds Sit to Stand; TUG: Timed Up and Go; ST: Step Test; 4StageBMT: Four-Stage Balance “Modified” Test; SEE: Self-Efficacy for Exercise Scale; PAPM: Activity and Participation Profile Related to Mobility.

## Data Availability

Data are contained within the article and Supplementary Materials.

## Supplementary Materials

The following supporting information can be downloaded at https://www.mdpi.com/article/10.3390/s26144616/s1: Table S1. ANCOVA analyses of post-intervention outcomes adjusted for baseline performance and age.
