# Supplementary material for: Exergaming for Healthy Aging: Associations with Functional Capacity, Social Participation, Self-Efficacy for Exercise, and Adherence to Inform Exergame Development
Source: Sensors (Basel). 2026 Jul 21;26(14):4616. doi: 10.3390/s26144616 (PMC13418576; doi:10.3390/s26144616)
Supplement: Supplementary file 1 [file sensors-26-04616-s001.zip › sensors-4323313-supplementary.pdf]

Supplementary Material

Supplementary Material Table S1. ANCOVA analyses of post-intervention outcomes adjusted for baseline performance and age

Outcome	n (EG/CG)	Adjusted mean EG (95% CI)	Adjusted mean CG (95% CI)	Adjusted mean difference (95% CI)	F	p	Partial η^2
**HS**	21/21	14.64 (12.90–16.38)	14.43 (12.69–16.17)	0.21 (−2.32 to 2.74)	0.029	0.866	0.001
**10-MWT**	14/7	0.784 (0.656–0.911)	0.575 (0.384–0.765)	0.209 (−0.035 to 0.454)	3.255	0.089	0.161
**30s STS**	27/18	9.27 (8.08–10.46)	7.32 (5.86–8.78)	1.95 (0.04–3.86)	4.260	**0.045**	0.094
**TUG**	26/13	17.96 (15.25–20.68)	24.63 (20.77–28.49)	−6.67 (−11.43 to −1.91)	8.102	**0.007**	0.188
**ST**	20/18	8.18 (6.93–9.42)	6.42 (5.10–7.73)	1.76 (−0.11 to 3.63)	3.680	0.063	0.098
**4-StageBMT**	27/20	4.53 (4.10–4.96)	3.64 (3.13–4.14)	0.89 (0.22–1.57)	7.082	**0.011**	0.141
**SEE**	27/20	14.73 (13.15–16.32)	12.86 (11.01–14.72)	1.87 (−0.62 to 4.37)	2.293	0.137	0.051
**PAPM**	13/13	0.881 (0.497–1.265)	1.861 (1.477–2.245)	−0.980 (−1.575 to −0.386)	11.710	**0.002**	0.347

HS: Handgrip Strength; 10-MWT: 10-Meter Walking Speed; 30s STS: 30 Seconds Sit to Stand; TUG: Timed up and Go; ST: Step Test; 4StageBMT: 4-Stage Balance "Modified" Test; SEE: Self-Efficacy for Exercise Scale; PAPM: Activity and Participation Profile related to Mobility. Note1: ANCOVA adjusted for age and the corresponding baseline value of each outcome measure. Adjusted means represent estimated marginal means evaluated at the mean values of the covariates. F, p values and partial η^2 correspond to the effect of group after adjustment. Note2: Post-intervention scores were entered as dependent variables, intervention group as the fixed factor, and age together with the corresponding baseline value of each outcome as covariates. Reported adjusted means correspond to estimated marginal means (EMMeans).
